# Family history of cancer and risk for esophageal and gastric cancer in Shanxi, China

**DOI:** 10.1186/1471-2407-9-269

**Published:** 2009-08-05

**Authors:** Ying Gao, Nan Hu, XiaoYou Han, Carol Giffen, Ti Ding, Alisa Goldstein, Philip Taylor

**Affiliations:** 1 Division of Cancer Epidemiology and Genetics, National Cancer Institute, Bethesda, MD 20852, USA; 2 Shanxi Cancer Hospital, Taiyuan, Shanxi 030013, PR China; 3 Information Management Services, Inc., Silver Spring, MD 20904, USA

## Abstract

**Background:**

Family history (FH) by different relative types and risk of upper gastrointestinal (UGI) cancers has been only rarely reported; the data on UGI cancer survival are sparse.

**Methods:**

600 esophageal squamous cell carcinoma (ESCC) cases, 598 gastric cardia adenocarcinoma cases, and 316 gastric non-cardia adenocarcinoma cases, and 1514 age-, gender-, and neighborhood-matched controls were asked for FH in first degree relatives and non-blood relatives. Odds ratios (ORs) and 95% confidence intervals (CIs) from logistic regressions, and hazard ratios (HRs) from Cox proportional hazard regressions were estimated.

**Results:**

Increased ESCC risk was associated with FH of any cancer (OR = 1.72, 95% CI = 1.39–2.12), FH of any UGI cancer (OR = 2.28, 95%CI = 1.77–2.95) and FH of esophageal cancer (OR = 2.84, 95%CI = 2.09–3.86), but not FH of non-UGI cancer. Individuals with two or more affected first-degree relatives had 10-fold increased ESCC risk. FH of gastric cardia cancer was associated with an increased risk of all three cancers. Cancer in non-blood relatives was not associated with risk of any UGI cancer. FH of UGI cancer was associated with a poorer survival rate among younger ESCC cases (HR = 1.82, 95%CI = 1.01–3.29).

**Conclusion:**

These data provide strong evidence that shared susceptibility is involved in esophageal carcinogenesis and also suggest a role in prognosis.

## Background

Esophageal cancer is the sixth most common cause of cancer death worldwide and the fourth most common malignancy in China [1]. Shanxi Province in north central China has among the highest esophageal cancer rates in the world [2]. Esophageal cancers in Shanxi are predominantly squamous cell carcinomas and adenocarcinomas are rare. The disease progresses rapidly; even when the tumors are surgically removed, the 5-year survival rate is less than 18% http://www.cancer.org/docroot/cri/content/cri_2_4_1x_what_are_the_key_statistics_for_esophagus_cancer_12.asp. However, the etiology of this disease is still largely unknown.

Studies have implicated a number of environmental exposures and predisposing conditions, predominantly tobacco smoking and alcohol drinking as risk factors of esophageal cancer in the Western world [3-5], although risk from these factors is either small or null in the especially high-risk populations of China and Iran [6-8]. Inherited susceptibility is also a very important factor in esophageal carcinogenesis, as suggested by familial aggregation [9-13], family history (FH) of cancer [3,14-19], segregation studies [20,21], and candidate gene association studies [22-25]. However, co-occurrence of esophageal cancer among family members does not necessarily reflect shared genetic susceptibility; it could also be due to shared environmental exposures. Therefore, studying different types of relatives (blood and non-blood relatives sharing household) might provide information to help differentiate genetic and environmental components in esophageal pathogenesis. To date, systematic exploration for the role of FH by relative type in esophageal cancer development has rarely been reported [16].

It has been suggested that familial esophageal cancer may develop earlier and have a poorer prognosis than sporadic esophageal cancer [26]. Therefore, it is reasonable to hypothesize that FH of cancer might also predict survival of upper gastrointestinal (UGI) cancer. The limited data available on esophageal cancer[26], gastric cancer [27,28] and colorectal cancer[29] on this are, however, inconclusive.

To examine the role of FH in esophageal cancer, we took advantage of a case-control study conducted in Shanxi, where the rates for both esophageal squamous cell carcinoma and gastric cardia adenocarcinoma are among the highest in the world [2]. Cancers at these two sites share some etiologic risk factors, and historically were diagnosed as a single disease referred as "hard swallowing disease" [30]. Therefore, we also evaluated the association between FH of cancer and gastric cancer risk, including gastric cardia cancer and gastric noncardia cancer. In addition, we examined the survival status of UGI cancer patients in relation to FH of UGI cancer.

## Methods

Patients presenting to the Shanxi Cancer Hospital in Taiyuan, Shanxi, People's Republic of China between 1997 and 2005 were potentially eligible for inclusion in this case-control study of upper gastrointestinal (UGI) tract cancers. The Shanxi Cancer Hospital, the largest cancer hospital in Shanxi, performed surgery on approximately 2000 new esophageal and 1800 new gastric cancers annually during the study period. We included cases in this study who: (i) were males or females 20 years of age or older, (ii) resided in one of five geographic regions in relatively close proximity to the hospital (Taiyuan, Linfen, Jinzhong, Chanzi, and Xinzhou), (iii) had newly diagnosed (incident) cancer of the esophagus or stomach without previous treatment (ie, no surgery, chemotherapy, or radiotherapy), (iv) underwent complete surgical resection of their tumors (ie, either esophagectomy or gastrectomy with curative intent, without neoadjuvant or adjuvant therapy) at the Shanxi Cancer Hospital, and (v) had their diagnosis histologically confirmed. During the study period, about two-thirds of new UGI cancers presenting to the Shanxi Cancer Hospital came from the five geographic regions we designated. Since one objective of our study was to evaluate somatic changes in tumors in UGI cancer cases, we limited recruitment to patients who had surgical resection of their tumor as their primary therapy. We invited a systematic sample (eg, all patients from selected days of selected weeks) of new UGI cancer patients from our designated geographic regions who underwent surgical resection (approximately 50% of such patients from these regions) to join the study; 98% of invitees accepted enrollment in the study.

Esophageal cancer cases were limited to those with histological esophageal squamous cell carcinoma (ESCC), which included nearly all esophageal cancers since adenocarcinoma of the esophagus is essentially nonexistent in this high-risk population. Gastric cardia adenocarcinoma (GCA) included adenocarcinomas located in the top three centimeters of the stomach, while gastric non-cardia adenocarcinoma (GNCA) included gastric cancers located in the remainder of the stomach. All histological diagnoses were made initially by pathologists at the Shanxi Cancer Hospital and confirmed by pathologists at the National Cancer Institute.

One control was enrolled for each case matched on age (± 5 years), gender, and neighborhood of residence. To identify potential controls, each case was asked to identify a neighbor of approximately the same age and gender. When the initial suggested neighbor could not be enrolled (i.e., unavailable, ineligible, or refused), other neighbors, or the village doctor were asked to suggest another neighbor of the same age and gender. Potential controls were asked if they had any cancer or UGI disease, and were considered ineligible if they reported affirmatively to either question. In addition, the interview for the control had to be completed within six months of its matched case to be included. Over 75% of all identified potential controls were enrolled, including 95% of available and eligible controls (i.e., the ones actually invited). The primary reason for non-enrollment among available/eligible controls was refusal to give a blood sample.

After obtaining informed consent, both cases (in hospital) and controls (at home) were interviewed to obtain information on demographic characteristics, lifestyle, and FH. Questionnaire-based information on occupation, education, tobacco smoking, alcohol use, and FH of cancer, were collected. For FH of cancer, questions were asked about any malignant tumor in first degree relatives, including father, mother, siblings, and offspring. In addition, information on cancer history was also collected on non-blood relatives in the same household as the cases and controls (i.e., spouses, adopted parents, step-parents, and adopted siblings).

In addition to information collected at the time of recruitment, cancer cases (or their immediate family) were re-contacted by study research nurses to determine their vital status and inquire about post-surgical treatment (i.e., chemotherapy, radiotherapy, traditional Chinese herbs, and other treatment) through the end of 2005. Deaths were recorded; cases still alive were censored as of the date of their last contact.

Since there were no malignant tumors reported in offspring, analyses of FH in first degree relatives were restricted to FH in father, mother, and siblings (both full and half -sibling). When numbers permitted, FH of esophageal cancer among first degree relatives was also examined separately by relation type (i.e., mother, father, and siblings). Cancers in spouses and other non-blood relatives in the same household were uncommon and were combined into a single group (i.e., non-blood relative) for analyses. The frequency distribution of siblings was the same for ESCC, GCA, and GNCA cases, and controls (median = 3, inter-quartile range = 2–5).

Odds ratios (ORs) and 95% confidence intervals (CIs) were calculated from logistic regressions. We confirmed that the three controls groups (for ESCC, GCA, and GNCA, respectively) did not differ significantly by the distribution of FH of cancer. To optimize power, all analyses used unconditional logistic regression adjusted for matching factors and pooled all controls. Geographic region was used as a surrogate for the neighborhood matching factor in unconditional logistic regression models. Adjustment for tobacco smoking, alcohol consumption, occupation, source of drinking water, scalding-hot food consumption, tea consumption, and number of siblings did not modify the results substantially. In particular, the ORs for ever tobacco smoking were 1.19 (95% CI 0.84–1.69), 0.80 (95% CI 0.60–1.06), and 1.12 (95% CI 0.74–1.69) for ESCC, GCA and GNCA respectively. Furthermore, the ORs for ever alcohol drinking were 1.18 (95% CI 0.93–1.50), 0.90 (95% CI 0.80–1.23), and 1.24 (95% CI 0.93–1.67) for ESCC, GCA and GNCA respectively. Therefore, only results adjusted for the matching factors (age, gender, and geographic region) are reported.

Hazard ratios (HRs) and 95% CIs from Cox proportional hazard regressions were calculated to estimate the association of FH in first degree relatives and survival time from UGI cancers. Survival time was calculated as days from UGI cancer surgery to death or date of last contact. Survival analyses were adjusted for matching factors and clinical characteristics of the tumor, including histological grade (well differentiated: G1 and G2; poor differentiated: G3 and G4), tumor stage (early: TIS, T1, and T2; late: T3 and T4) and lymph node metastasis (yes vs. no). In addition, we also conducted analyses by further adjusting for post-surgical treatment. Finally, we examined the ESCC survival in all cases, cases <50 years, and cases ≥50 years by family history of UGI cancer using Kaplan-Meier plots.

All analyses were two-sided, and statistical significance was defined as a P-value less than 0.05.

The study was approved by the institutional review boards of the Shanxi Cancer Hospital in Taiyuan, and the National Cancer Institute in Bethesda, Maryland.

## Results

A total of 600 ESCC, 598 GCA, and 316 GNCA cases, and 1514 age-, gender-, and neighborhood- matched controls were included in these analyses. All cases were histologically confirmed. Among the cases, 32 ESCC, 70 GCA, and 21 GNCA cases did not have follow-up data, resulting in 568 ESCC, 529 GCA, and 295 GNCA cases available for the survival analyses. Gender, age, and geographic region distributions of study subjects are shown in Table 1. Clinical characteristics of the cases available for the survival analyses are shown in Table 2. Overall, nearly three-quarters of cases were males, and the median age at diagnosis was 59 years. Most cancer cases were diagnosed at late stage. The median follow-up time was about 3 years and median survival time after surgery was about 2 years.

**Table 1:** Selected demographic characteristics of UGI cancer cases and controls

		Controls (N = 1514)	ESCC (N = 600)	GCA (N = 598)	GNCA (N = 316)
Gender	Male (%)	1107(73)	376(63)	491(82)	239(76)
	Female (%)	407(27)	224(37)	107(18)	77(24)

Age (median, inter-quartile) (years)		59(52–65)	58(51–64)	61(55–66)	57.5(50–63)
	Male	60(53–65)	59 (52–64)	61.5(55–66)	58(51–63)
	Female	57(50–63)	57(50.5–63)	60(54–64)	54(44–63)

Geographic regions	Taiyuan (%)	524(35)	212(35)	199(33)	113(36)
	Linfen (%)	266(18)	94(16)	118(20)	54(17)
	Jinzhong (%)	294(19)	153(26)	104(18)	37(12)
	Chanzi (%)	274(18)	90(15)	121(20)	63(20)
	Xinzhou (%)	156(10)	51(8)	56(9)	49(15)

**Table 2:** Selected clinical characteristics of UGI cancer cases

		ESCC (N = 568)	GCA (N = 529)	GNCA (N = 295)
Survival status	Deceased	345(61)	378(71)	194(66)
	Censored	223(39)	155(29)	101(34)

Histological grade	Well differentiated (G1 or G2)	450(79)	186(35)	78(27)
	Poor differentiated (G3 or G4)	118(21)	341(65)	215(73)

Primary tumor stage	Early(TIS or T1 or T2)	95(17)	26(5)	33(11)
	Late(T3 or T4)	473(83)	502(95)	261(89)

Lymph node metastasis	No	330(58)	140(27)	80(27)
	Yes	238(42)	386(73)	213(73)

Survival days from surgery	Median (inter quartile)	794(356–1979)	622(314–1606)	615(256–2147)

### FH of any malignant tumor and risk of UGI cancer

Subjects (i.e., cases and controls) reported the occurrence of 58 different types of malignant tumors in their family members. A FH of any malignant tumor in a first degree relative was associated with 1.72, 1.32, and 1.52-fold increased risks of ESCC, GCA, GNCA respectively (Table 3). Though not significant, the associations appeared stronger in males and younger persons (<50 years). Subjects with more than one cancer-affected first degree relative had higher risks of UGI cancers than those with only one affected relative (P trend < 0.01 each for ESCC, GCA, and GNCA). Cancers in non-blood relatives were not associated with risk of any of the UGI cancers evaluated here.

**Table 3:** Risk of UGI cancer by FH of malignant tumor in first degree relatives and non-blood relatives*

		ESCC		GCA		GNCA	
FH	Control (%)	Case (%)	OR (95% CI)	Case (%)	OR (95% CI)	Case (%)	OR (95% CI)
Any malignant tumor							
First degree relative	334(22)	197(33)	1.72(1.39–2.12)	162(27)	1.32(1.06–1.64)	94(30)	1.52(1.15–1.99)
							
1 affected	294(19)	160(27)	1.58(1.26–1.98)	135(22)	1.25(0.99–1.58)	79(25)	1.43(1.07–1.92)
≥ 2 affected	40(3)	37(6)	2.76(1.73–4.40)	27(5)	1.83(1.10–3.03)	15(5)	2.15(1.16–3.98)
P trend			<0.001		0.005		0.001
							
Male	233(21)	126(34)	2.00(1.50–2.54)	132(27)	1.35(1.05–1.73)	78(33)	1.83(1.34–2.49)
Female	101(25)	71(32)	1.40(0.98–2.02)	30(28)	1.24(0.76–2.01)	16(21)	0.81(0.44–1.48)
							
< 50 yr	59(22)	41(38)	2.17(1.32–3.56)	22(33)	1.95(1.06–3.58)	28(36)	2.39(1.35–4.25)
≥ 50 yr	275(22)	156(32)	1.61(1.27–2.04)	140(26)	1.25(0.98–1.58)	66(28)	1.35(0.98–1.85)
							
Non-blood relative	58(4)	26(4)	1.08(0.66–1.76)	25(4)	1.02(0.62–1.66)	6(2)	0.61(0.26–1.46)

Any UGI cancer							
First degree relative	170(11)	131(22)	2.28(1.77–2.95)	104(17)	1.62(1.24–2.12)	53(17)	1.65(1.17–2.33)
							
1 affected	162(11)	114(19)	2.08(1.59–2.71)	88(15)	1.43(1.08–1.90)	46(14)	1.50(1.05–2.15)
≥ 2 affected	8(1)	17(3)	6.37(2.82–15.6)	16(3)	5.35(2.26–12.7)	7(2)	4.78(1.68–13.6)
P trend			<0.001		<0.001		0.001
							
Male	124(11)	88(23)	2.59(1.90–3.54)	87(18)	1.66(1.23–2.23)	45(19)	1.89(1.29–2.77)
Female	46(11)	43(19)	1.85(1.16–2.92)	17(16)	1.52(0.82–2.80)	8(10)	0.98(0.43–2.20)
							
< 50 yr	34(12)	23(21)	1.84(1.01–3.35)	10(15)	1.23(0.56–2.68)	17(22)	2.34(1.18–4.65)
≥ 50 yr	136(11)	108(22)	2.37(1.78–3.16)	94(18)	1.70(1.28–2.27)	36(15)	1.51(1.01–2.26)
							
Non-blood relative	26(2)	12(2)	1.11(0.54–2.24)	13(2)	1.17(0.59–2.32)	3(1)	0.73(0.22–2.45)

Esophageal cancer							
First degree relative	97(6)	95(16)	2.84(2.09–3.86)	52(9)	1.34(0.94–1.91)	28(9)	1.56(0.99–2.43)
							
1 affected	93(6)	83(14)	2.53(1.84–3.50)	43(7)	1.15(0.79–1.68)	25(8)	1.44(0.90–2.30)
≥ 2 affected	4(0.3)	13(2)	10.0(3.24–31.2)	9(2)	5.75(1.74–19.0)	3(1)	4.54(0.96–21.6)
P trend			<0.001		0.022		0.026
							
Father	52(3)	40(7)	2.01(1.31–3.10)	23(4)	1.06(0.64–1.76)	13(4)	1.27(0.68–2.38)
Mother	29(2)	34(6)	3.27(1.96–5.47)	19(3)	1.69(0.93–3.07)	14(4)	2.52(1.30–4.89)
Sibling	21(1)	35(6)	4.66(2.67–8.13)	20(3)	2.36(1.26–4.41)	4(1)	1.14(0.38–3.40)
							
Non-blood relative	9(0.6)	7(1)	1.86(0.68–5.10)	6(1)	1.49(0.52–4.26)	1(0.3)	0.76(0.09–6.16)
							
Gastric cardia cancer	25(2)	23(4)	2.45(1.37–4.39)	28(5)	2.87(1.65–5.00)	12(4)	2.35(1.16–4.79)
							
Gastric noncardia cancer	51(3)	19(3)	0.95(0.56–1.64)	31(5)	1.54(0.97–2.44)	17(5)	1.52(0.86–2.69)

Any non- UGI tumor							
First degree relative	164(11)	66(11)	0.98(0.72–1.33)	58(10)	0.92(0.67–1.27)	41(13)	1.22(0.84–1.76)
Non-blood relative	32(2)	14(2)	1.05(0.55–2.01)	12(2)	0.90(0.46–1.77)	3(1)	0.54(0.16–1.80)

*The reference group for each analysis, the group of individuals with negative family history, is omitted from this table;

ORs adjusted for age, gender, and geographic region; First degree relatives: father, mother, and siblings; Non-blood relatives: spouse, adopted or step-parents, and adopted brother

### FH of UGI cancer and risk of UGI cancer

FH of any UGI cancer was associated with 2.28, 1.62, and 1.65-fold increased risks of ESCC, GCA, and GNCA (Table 3). Neither age nor gender modified these associations. Having two or more affected relatives showed higher risks of all three UGI cancers. In contrast, any UGI cancer in non-blood relatives was not associated with increased risk of ESCC, GCA, or GNCA.

FH of esophageal cancer was associated with an increased risk of ESCC, but not GCA or GNCA overall. However, increased risk for GCA and GNCA was observed among persons with more esophageal cancer-affected relatives. Though FH of gastric cardia cancer was associated with increased risk for all three UGI cancers (GCA, ESCC, and GNCA), FH of gastric noncardia cancer was not associated with risk of any UGI cancers evaluated.

FH of non-UGI cancer in either first degree relatives or non-blood relatives was not associated with risk of ESCC, GCA, and GNCA.

### FH of esophageal cancer by relative type and risk of UGI cancer

FH of esophageal cancer in first degree relatives was associated with a 2.84-fold increase of ESCC risk (Table 3). Neither age (P interaction = 0.91) nor gender (P interaction = 0.23) modified this association. While individuals with one affected first degree relative had a 2.53-fold increased risk of ESCC (95% CI = 1.84–3.50), those with two or more affected first degree relatives had a 10.0-fold increased ESCC risk (95% CI = 3.24–31.2) (P trend < 0.01). The ESCC odds ratios associated with father, mother, and sibling histories of esophageal cancer were 2.01, 3.27, and 4.66, respectively. The risk of GCA (P trend = 0.02) and GNCA (P trend = 0.03) increased monotonically with greater numbers of affected relatives. No association was seen between esophageal cancer in non-blood relatives and risk of ESCC, GCA, or GNCA.

### FH of malignant tumor and survival from UGI cancer

**Figure 1:**
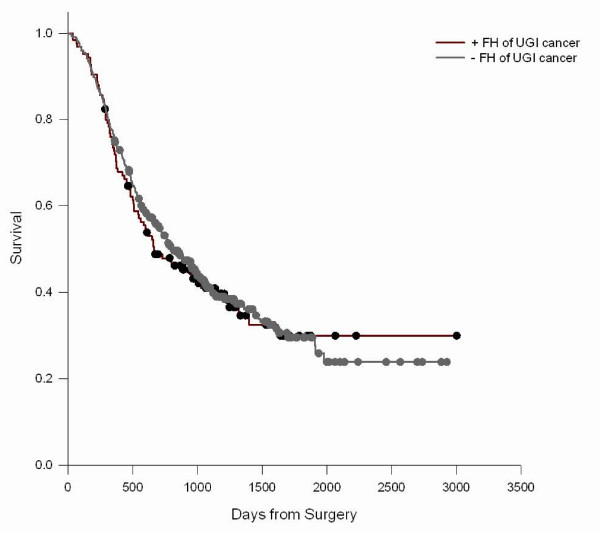
**ESCC survival in cases by FH of UGI cancer**. Log-Rank test P = 0.7286

**Figure 2:**
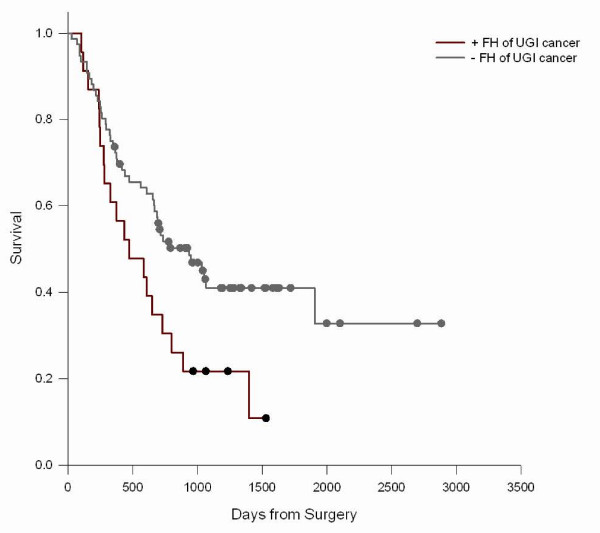
**ESCC survival in cases by FH of UGI cancer < 50 years old**. Log-Rank test P = 0.0.0228

**Figure 3:**
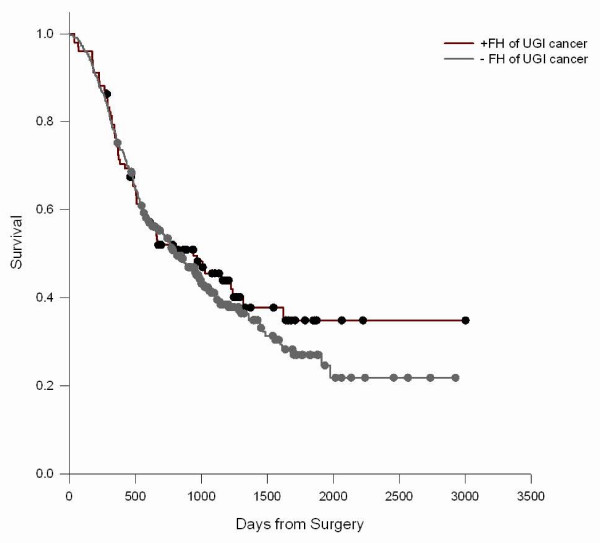
**ESCC survival in cases by FH of UGI cancer ≥ 50 years old**. Log-Rank test P = 0.4829

We did not observe significant associations between FH of any malignant tumor, any UGI cancer, or esophageal cancer, gastric cardia cancer, and gastric noncardia cancer in first degree relatives and survival from ESCC, GCA, and GNCA (Table 4). Though not significant, there was a suggestion of longer survival in GCA cases with a positive FH. The relation of FH to survival was modified by age (P interaction = 0.01) (Figure 1) such that younger (<50 years) ESCC cases with a positive FH had poorer survival (Figure 2), but not older cases (Figure 3); median survival for FH positive ESCC cases was 473 days versus 712 days for FH negative cases. A similar age-related pattern was also observed for GCA cases (P interaction = 0.06). FH of non-UGI cancer was associated with increased death rate for GNCA (HR = 1.73, 95% CI = 1.16–2.60).

**Table 4:** FH of malignant tumor in first degree relatives and survival from UGI cancer *

		ESCC			GCA			GNCA		
	FH	Case	Death	HR (95% CI)	Case	Death	HR (95% CI)	Case	Death	HR (95% CI)
Any malignant tumor	-	379	232	1	384	278	1	210	134	1
	+	190	113	1.09(0.86–1.36)	146	96	0.80(0.63–1.01)	85	60	1.22(0.88–1.68)

Any UGI cancer	-	443	268	1	434	309	1	245	164	1
	+	126	77	1.06(0.82–1.38)	96	65	0.82(0.62–1.08)	50	30	0.85(0.56–1.28)
										
0 affected	-	443	268	1	434	309	1	245	164	1
1 affected	+	109	69	1.15(0.88–1.50)	82	55	0.80(0.60–1.07)	43	23	0.73(0.46–1.15)
≥2 affected	+	17	8	0.64(0.32–1.30)	14	10	0.94(0.48–1.84)	7	7	1.82(0.84–3.97)
										
<50	+	23	19	1.82(1.01–3.29)	8	7	2.30(0.85–6.20)	16	8	0.96(0.38–2.38)
>=50	+	103	58	0.92(0.68–1.22)	88	58	0.76(0.57–1.02)	34	22	0.78(0.49–1.26)

Esophageal cancer	-	479	291	1	481	342	1	268	174	1
	+	90	54	0.97(0.72–1.31)	49	32	0.81(0.56–1.18)	27	20	1.03(0.63–1.68)
										
Gastric cardia cancer	-	546	327	1	504	356	1	284	189	1
	+	23	18	1.61(0.99–2.61)	26	18	0.88(0.54–1.42)	11	5	0.66(0.26–1.64)
										
Gastric noncardia cancer	-	550	336	1	503	356	1	279	185	1
	+	19	9	0.80(0.41–1.56)	27	18	0.76(0.47–1.22)	16	9	0.96(0.48–1.91)

Non-UGI cancer	-	505	309	1	480	343	1	260	164	1
	+	64	36	1.10(0.77–1.56)	50	31	0.82(0.56–1.20)	35	30	1.73(1.16–2.60)

*HRs adjusted for age, gender, geographic region, histologic grade, primary tumor stage, and lymph node metastatsis

In addition, we also conducted analyses by further adjusting for post-surgical treatment. However, the results were not substantially modified: HRs of ESCC, GCA, and GNCA cases with FH of any UGI cancer were 1.04(0.80–1.34), 0.82(0.62–1.08), and 0.84(0.56–1.26) respectively; HRs of ESCC, GCA, and GNCA cases with FH of esophageal cancer were 0.96(0.71–1.28), 0.81(0.56–1.18), and 1.05(0.65–1.72), respectively. Therefore, only results without post-surgical treatment adjustment were reported.

## Discussion

In this large case-control study focusing on FH of malignant tumors and risk of UGI cancers, we observed increased risk for ESCC among individuals with FH of any malignant tumor, any UGI cancer, and esophageal cancer. The association strengthened as FH became more organ-specific, and was strongest for a FH of esophageal cancer. FH of non-UGI cancer was not associated with ESCC risk, suggesting that the increased risk for FH of any malignant tumor was due to UGI cancer. Risk also increased with greater number of affected relatives. While FH of gastric cardia cancer was associated with an elevated risk of ESCC, GCA, and GNCA, FH of gastric noncardia cancer did not affect risk. Notably, cancers in non-blood relatives were not associated with risk of any UGI cancers evaluated. FH did not predict prognosis among cases overall, but was suggestively associated with reduced death rate from GCA, and younger ESCC cases with a FH of UGI cancer experienced poorer survival.

The positive association between FH of esophageal cancer and ESCC risk in the current study is consistent with previous reports [3,6,14-16,19,31-36], and suggests shared susceptibility, including both genetic and environmental factors, in esophageal cancer pathogenesis.

Aggregation of cancer among non-blood relatives in the same household, like wives and husbands, supports environmental compared to genetic factors in cancer etiology. A cohort study from Japan examined the correlation between questionnaire-based history of cancer in fathers and mothers of cohort members, and observed more parent pairs with esophageal cancer, as well as stomach cancer, than expected [37], consistent with a role for environmental factors in these cancers. In contrast, a case-only study of esophageal cancer that examined FH of esophageal cancer by relative type found more esophageal cancer patients among blood relatives than non-blood relatives [17]. Our findings are more consistent with the latter study since we found significant effects only for blood relatives. Further, the stronger ORs and dose response pattern seen for multiple affected relatives suggests a role for genetic susceptibility in etiology[6]. Since we have no information on environmental exposures in blood or non-blood relatives, we can not evaluate environmental factors in the observed familial aggregation.

The ESCC risk estimates showed consistent increases with FH of esophageal cancer in fathers, mothers, or siblings in the current study, with the strongest association with siblings. Our risk estimate for sibling history was nearly identical to the report of Garavello et.al. (OR = 4.6, 95% CI = 1.2–17.4).^13^, and is consistent with recessive heredity, or a gene dose effect involved in the pathogenesis of esophageal cancer [38]. However, we can not exclude the possibility of shared environment exposures in childhood [39], a critical period for cancer development.

Some studies suggest that FH increases the risk of cancer at many sites and is not site-specific [40]. The association between FH and ESCC risk in our study strengthened as FH became more specific: OR = 1.48 for FH of any cancer, OR = 1.66 for FH of any UGI cancer, and OR = 1.99 for FH of esophageal cancer. In contrast, no association was observed between FH of non-UGI cancer and risk of ESCC. This suggests that the shared susceptibility is UGI-specific, predominantly esophagus-specific, and FH of non-UGI cancer is not important for UGI cancer risk prediction in this population. A similar pattern was observed for risk of GCA and GNCA.

Younger age at onset is often taken to suggest a genetic role in disease. In a previous case-control study [26], FH correlated with early age of onset for esophageal cancer. Similarly, a small case-control study reported higher risk among younger people who had a FH [16]. We explored age at onset but were not able to detect any significant age effects.

The majority of previous studies reported increased risk of gastric cancer among people with a positive FH [15,41-50]. In the only two studies that examined the risk of gastric cancer by anatomic sub sites (cardia and non-cardia) [47,50], FH of gastric cancer was associated with increased risk of non-cardia cancer only [50], and FH of any cancer was associated with increased risk of cardia cancer only [47]. With more detailed information in the current study, we observed that FH of gastric cardia cancer was associated with increased risk for GCA as well as GNCA, but no association was observed between FH of gastric noncardia cancer and risk of either GNCA or GCA.

Since the esophagus and stomach are anatomically adjacent, they may share some common etiological factors. With the detailed FH information collected here, we were able, for example, to evaluate FH of esophageal cancer as a risk factor for both ESCC and GCA, as well as FH of gastric cardia cancer as risk factor for ESCC and GCA. Consistent with previous reports, we did not observe an overall association between FH of esophageal cancer and risk of GCA or GNCA [15,42]. However, we observed an increased risk of GCA and GNCA in persons with more esophageal cancer-affected relatives, which suggests shared susceptibility among these cancers. Alternatively, we cannot rule out misclassification since no FH cancer reports were confirmed. Two studies that examined the relationship between FH of gastric cancer and ESCC risk found null results [15,34]. In contrast, we observed an increased ESCC risk with FH of gastric cardia cancer but not FH of non-cardia cancer. Differences from previous reports could be due to variation in the prevalence of gastric cardia adenocarcinoma in the study populations. Moreover, even though our study is large, FH was based exclusively on self reports, and differences could also be due to miscategorization.

Several lines of evidence support a role of genetics in cancer survival: FH of colorectal cancer is associated with improved survival in stage III colon cancer [29]; gene polymorphisms have been related to differences in survival of breast cancer cases [51,52]; and familial UGI cancer cases have shown higher microsatelite instability [53], greater loss of heterozygosity [54], and different gene expression patterns from sporadic cases [55]. Therefore, it is reasonable to hypothesize that FH might affect both risk and prognosis in UGI cancers. A large study with 1715 ESCC cases conducted in Hebei by Wen and colleagues found poorer prognosis in ESCC patients with a FH of UGI cancers [26]. Though we did not find an association between FH of cancer and survival from ESCC overall, survival was poorer in young cases with a positive FH. These results were not substantially modified by additional adjustment for post-surgical treatment. This provides some evidence that familial esophageal cancers have an altered clinical course from sporadic cases, an observation which needs confirmation in other studies.

A number of studies have examined FH in relation to gastric cancer survival [27,28,56,57], but only one reported a suggestively reduced death rate (HR = 0.82, 95% CI = 0.62–1.08) for cases with a positive FH [27]. We observed a similar, statistically insignificant, reduced death rate (HR = 0.82, 95% CI = 0.62–1.08) in GCA cases, but not GNCA cases. These suggestions of improved survival status among GCA cases may indicate the existence of different pathways for the progression of familial cancer from sporadic cancer. Though the reason is unclear, we also observed an increased death rate among GNCA cases with a FH of non-UGI cancer.

There are several advantages to our study: large sample size, high participation rates for cases (100%) and controls (95%), high follow-up rate (over 90%), neighborhood-matched controls, detailed information on FH of any malignant tumor, and cancer information from non-blood relatives. These advantages allowed us to explore site-specific familial aggregation of cancer and differentiate roles of genetic and environmental factors in UGI cancer development. Limitations of this study are: potential recall bias due to the nature of the case-control study design, under-ascertainment of FH, limited confirmation of cancer reports from relatives, potential misclassification of cancer types in relatives, and limited number of families with multiple esophageal cancer-affected members in ESCC controls, as well as small number of non-blood relatives with cancers.

## Conclusion

In this large study focused on FH and UGI cancer, we observed an increased ESCC risk among individuals with a FH of malignant tumors. The association was esophagus-specific, and stronger with multiple affected relatives. Cancers in non-blood relatives were not associated with UGI cancer risk. FH of UGI cancer was also associated with poorer survival rate in young ESCC cases. These data provide strong evidence that shared genetic susceptibility is involved in esophageal carcinogenesis and also suggest a role in prognosis.

## Competing interests

The authors declare that they have no competing interests.

## Authors' contributions

PT, NH, AG, and CG designed the study. XH and TD collected the data. YG and PT analyzed data and wrote the report. All authors read, gave comments, and approved the final version of the manuscript. YG and PT had full access to all of the data in the study and take responsibility for the integrity of the data and the accuracy of the data analysis.

All authors read and approved the final manuscript.

## Pre-publication history

The pre-publication history for this paper can be accessed here:

http://www.biomedcentral.com/1471-2407/9/269/prepub
